# Leukæmia in a Cat

**Published:** 1896-05

**Authors:** Wilfried Lellmann

**Affiliations:** Professor at New York College of Veterinary Surgeons


### LEUKÆMIA IN A CAT.

By Dr. WILFRIED LELLMANN,
PROFESSOR AT NEW YORK COLLEGE OF VETERINARY SURGEONS.

During the end of February I was requested to examine a cat, which according to the history of the case had been falling away for several weeks, although the appetite had always been quite good. A thorough examination of the patient gave the following results:

White female cat, about two to three years of age, in a poor state of nutrition, staring coat, hair lustreless, erected, showing general weakness. The visible mucous membranes were remarkably pale; the pulse was rather small, soft, and irregular, beating about 150 per minute. The heart-beating could be felt on both sides of the thorax very distinctly, and was rather a little stronger than normal. As the animal was in an emaciated condition the heart-shock could be seen on the left side of the thorax. Percussion of the heart-region gave enlarged dulness of the heart, the sound being somewhat tympanitic. When auscultating the heart I heard both tones, the first being somewhat higher (metallic timbre); the second tone weaker and not to be heard very distinctly. A sound was to be heard besides synchronical with the systole. This, however, was not to be referred to an organic trouble; it was simply a so-called anorganic noise (abnormal bruits of anæmia). According to the above symptoms of the circulatory apparatus I diagnosed dilatation of the heart.

The temperature, measured in the rectum, was $102\frac{2}{5}$°. The respiration was increased to 48 per minute. The percussion of the thorax did not give anything abnormal, except the above-mentioned dulness of the heart. The auscultation of the lungs did not prove anything abnormal; vesicular breathing, although a little sharp, was heard. Appetite was good; feces looked dark and were rather thin. When palpating the abdomen, the spleen was felt distinctly, and seemed to be enlarged; also the liver, the submaxillary, post-pharyngeal, supramammary, and inguinal glands could be pointed out easily; they were also enlarged. Urine could not be obtained, although I was very anxious to make an analysis and microscopical examination of the same.[1] The physical behavior of the cat seemed to be perfectly normal; she was comparative lively.

In order to complete the diagnosis I proceeded to make an examination of the blood. This was drawn from the ear and examined by means of the Thoma-Zeiss hæmacytometer. Macroscopically the blood appeared to be somewhat discolored, having a dirty reddish color and coagulating rather slowly. Both red and white blood-corpuscles were counted several times; the counting resulted in the proportion of 1 : 12, while the normal proportion is 1 white corpuscle to 350 red blood-corpuscles. While making the examination of the blood I found among the numerous leucocytes principally the multi-nuclear cells, also some mononuclear and some in hyaline stage. A great many red blood-corpuscles in the embryonic stage were also found. Toikilocythosis was also to be seen. The diagnosis was now assured as *leukœmia*. As is well-known we distinguish three different forms, that is, lymphatic, lienal, and myelogenic form. In this case I diagnosed the presence of all three forms. I based the myelogenic form upon the presence of a great many red blood-corpuscles in the embryonic stage.

The condition of the patient, which had been the same for about one week, now changed suddenly, the animal showing a rapid collapse, and died under the symptoms of internal hemorrhages. An autopsy was made immediately after death and proved the diagnosis to be correct.

Autopsy: When removing the skin a subcutaneous hemor-

[1] It would have been interesting to make an analysis of the urine, as we know that the so-called alloxur bodies (uric acid, xanthin, hypoxanthin, guanin, carnin, hetero-xanthin, etc.) are increased in leukæmia. This is to be explained by the decomposition of the multiplied leucocytes.

rhage was found on the left side of the thorax, measuring about 1½ tablespoonfuls. The abdominal cavity contained a serous liquid of 2 tablespoonfuls. The peritoneum was of grayish-white color. The intestinal canal was almost empty, having only very little liquid contents and half a dozen ascarides. The solitary follicles of the large intestines were enlarged. The rectum contained a little feces of soft consistency. The liver was enlarged, and showed on the diaphgramatic surface of the lower portion of the medial right lobes small hemorrhages under the serous membrane. On section the acini were found enlarged, but could not be distinctly recognized, the liver-tissue being of grayish-yellow color; the central veins hardly contained any blood; the consistency of the liver was rather soft. The spleen was enlarged, of rather hard consistency, and of dark-red color. The Malpighian bodies could be distinctly recognized, and appeared to be larger than normal. The mesenteric glands, the pancreas Aselli, and the inguinal glands were considerably enlarged, some of them having the size of a large bean. On section the kidneys showed small hemorrhages in the pyramids, otherwise normal. The pleural cavity contained a bloody exudate of about one tablespoonful. Heart was much enlarged, the pericardium containing half a teaspoonful of a bloody exudate. The heart was in diastole, both ventricles containing blood, the right ventricle more than the left. The heart was very much dilated, and the walls of the right ventricle very thin; the walls of the left ventricle were also atrophied. The atrio-ventricular and semilunar valves were normal. The heart-muscle itself showed a somewhat grayish-red color, being dry on section. The lungs were not entirely retracted. On the dorsal surface of the visceral pleura a great many small white spots were to be seen, varying in size from a pin-head to that of a grit-granule. On section a foamy fluid could be scraped from the surface. The bronchial and mediastinal lymphatic glands were also enlarged. The consistency of all the lymphathic ganglions was rather soft; on section they showed a grayish color. The cerebral and spinal meninges showed numerous very small hemorrhages. The bones of the extremities were examined, the marrow being of a rather dark, red color and gelatinous consistency, in short having the appearance of raspberry jelly. The tela ossea was rather thin, the bones being frangible.

